# Circulating miR-30a, miR-126 and let-7b as biomarker for ischemic stroke in humans

**DOI:** 10.1186/1471-2377-13-178

**Published:** 2013-11-16

**Authors:** Guangwen Long, Feng Wang, Huaping Li, Zhongwei Yin, Chaugai Sandip, Yan Lou, Yan Wang, Chen Chen, Dao Wen Wang

**Affiliations:** 1 Department of Internal Medicine and the Institute of Hypertension, Tongji Hospital, Tongji Medical College of Huazhong University of Science and Technology, Wuhan 430030, People’s Republic of China

**Keywords:** Circulating miRNA, Biomarker, Stroke

## Abstract

**Background:**

Recently, plasma miRNAs have been reported as biomarkers for various diseases. However, the knowledge on the association of plasma miRNAs with ischemic stroke is still lacking. In this study, we investigated whether plasma concentrations of miR-30a, miR-126 and let-7b may be biomarkers for ischemic stroke in humans.

**Methods:**

One hundred ninety seven patients with ischemic stroke were recruited and their blood samples were collected at 24 h, 1 week, 4 weeks, 24 weeks and 48 weeks after symptoms onset, and fifty healthy volunteers were selected as control. Levels of miRNA were quantified by quantitative real-time PCR. Relative expression level of miRNA was calculated using 2^-ΔΔct^ method. The ability to distinguish the ischemic stroke group from control group was characterized by receiver operating characteristic (ROC) curve, and the area under ROC curve (AUC) was calculated.

**Results:**

Circulating miR-30a and miR-126 levels were markedly down-regulated in all patients with ischemic stroke until 24 weeks. However, circulating let-7b was lower in patients with large-vessel atherosclerosis than healthy volunteers, whereas circulating let-7b had higher level in patients with other kinds of ischemic stroke until 24 weeks. Among all patients, circulating miRNAs levels returned to normal 48 weeks after symptom onset. Receiver operating characteristic (ROC) curve analysis showed that the areas under the curve (AUC) of plasma miR-30a were 0.91, 0.91, 0.92 and 0.93, the miR-126 were 0.92, 0.94, 0.93 and 0.92, and let-7b were 0.93, 0.92, 0.92 and 0.91 at 24 h, 1 w, 4 w and 24 w, respectively.

**Conclusions:**

These data suggest that miR-30a, miR-126 and let-7b might be useful biomarkers for ischemic stroke in humans.

## Background

Stroke is a leading cause of death and long-term disability in developed countries, and ~80% of strokes are ischemic in origin [1]. In China, 2.5 million people have stroke and 1 million die from stroke-related causes every year [2]. Multiple risk factors for stroke include advanced age, diabetes mellitus, hypercholesterolemia, hypertension, alcohol, smoking etc. [3].

MicroRNAs (miRNAs) are a novel family of non protein-coding short RNA molecules that regulate gene expression by recognizing binding sites located in the 3′ untranslated region (3′ UTR) of mRNA targets [4,5]. MiRNAs participate in a large number of physiological and pathological processes, such as differentiation, development, proliferation, apoptosis and migration [6-8]. However, compared with oncology or cardiology researches, a few studies have investigated the roles of miRNAs in neuronal death, degeneration or ischemic stroke [9-11]. For instance, progressive neurodegeneration occurs in the absence of Dicer, which is the crucial regulator of miRNA biogenesis, and miR-8 targets atrophin to prevent neurodegeneration in Drosophila [12]. The miR-146aG allele and miR-146aG/-149 T/-196a2C/-499G allele combinations were found to be associated with ischemic stroke pathogenesis [13]. MicroRNA-195 protects against dementia induced by chronic brain hypoperfusion via its anti-amyloidogenic effect in rats [14].

The involvement of miRNA in regulating the pathogenesis associated with middle cerebral artery occlusion (MCAo) in SD rats was first reported by Jeyaseelan et al., which demonstrated that miR-30a-3p was down-regulated in the 24-hour-reperfused MCAo rat brains but was subsequently up-regulated during the 48-hour reperfusion [15]. Recent studies indicate that miR-30 family regulates angiogenesis [16], and endothelium specific miRNA--miR-126 was down-regulated in young stroke patients [17]. Moreover, the expression of LIN28B and let-7 miRNA correlated with rs17065417 genotype in neuroblastoma cell lines [18]. Let-7 activates Toll-like receptor 7 that contributes to the spread of CNS damage [19].

Acute myocardial ischemia and ischemic stroke have similar pathophysiology, and our previous studies implied that the plasma concentration of miRNAs can be potential indicators of AMI [20-22]. Using the levels of circulating miR-30a, miR-126 and let-7b at early phase of AMI, we were able to define a score with a high sensitivity and specificity for the detection of AMI patients [21,22].

However, it is not clear whether miR-30a, miR-126 and let-7b are involved in ischemic stroke and specifically, assosiation of their plasma levels and ischemic stroke has not been reported. In the present study, we assessed the hypothesis that circulating miR-30a, miR-126 and let-7b might be useful for identifying and evaluating ischemic stroke in humans.

## Methods

### Blood samples

Experiments were conducted in accordance with the principles of Declaration of Helsinki. This study was approved by the Ethics Committee of Tongji Hospital. Written informed consents were obtained from all the participants and 247 blood samples (5 ml) were collected from the ischemic stroke patients and healthy volunteers at Tongji hospital from June 2009 to October 2009. The study included first-ever stroke patients with cerebral infarction. Diagnosis was based on the International Classification of Diseases, Ninth Revision as described previously [23]. Imaging studies were reviewed by experienced neuroradiologists to confirm the diagnosis and identify the stroke subtypes.

The ischemic stroke patients identified by World Health Organization clinical criteria were further classified according to TOAST classification, a) large-vessel atherosclerosis (LA, n = 51); b) small-vessel disease (SA, n = 48); c) cardioembolism (CEmb, n = 50); d) undetermined cause (UDN, n = 48) [17]. The patients’ functional status at the time of blood sampling was evaluated with the modified Rankin Scale (mRS). Exclusion criteria included other types of stroke (transient ischemic attack, subarachnoid hemorrhage, embolic brain infarction, brain tumors, and cerebrovascular malformation); severe systemic diseases, for example, pulmonary fibrosis and endocrine and metabolic diseases (except type 2 diabetes); inflammatory and autoimmune diseases; and serious chronic diseases, for example, hepatic cirrhosis and renal failure. Subjects with cardioembolic stroke and documented atrial fibrillation were also excluded from the study. In addition, healthy volunteers meeting the same exclusion criteria as the cases (negative imaging studies and no history of cerebrovascular disease, n = 50) were enrolled in our study.

This study was a cross-sectional study, stroke patients were recruited at different time points after stoke, and each time point was represented by a different set of patients. The blood samples of patients with ischemic stroke were obtained at 24 h (within 24 h), 1 w (±24 h), 4 w (±24 h), 24 w (±48 h) and 48 w (±72 h) after the onset of symptoms. Plasma was isolated by centrifugation and was maintained at −80°C until purification.

### RNA purification

We extracted total RNA from plasma with TRIzol LS Reagent as described previously [21].

### miRNA qRT-PCR

Two μg of total RNA were reverse transcribed by Transcript First-strand cDNA synthesis superMix (TransGen Biotech, Beijing, China) according to the manufacturer’s protocol. In brief, 50 μl reactions were incubated for 60 min at 42°C, 10 min at 70°C, and then preserved at 4°C.

qRT-PCR were performed using the Bulge-Loop™ miRNA qRT-PCR Detection Kit (Ribobio Co., Guangzhou, China) and *TransStart*™ Green qPCR SuperMix (TransGen Biotech, Beijing, China) according to the manufacturer’s protocol with the Rotor-Gene 6000 system (Corbett Life Science, Qiagen, Hilden, Germany). In brief, the reaction was incubated at 95°C for 30 s, and followed by 40 cycles of 95°C for 30 s, 60°C for 20 s, 70°C for 1 s. The relative expression level of each miRNA was calculated using the comparative CT method. MiRNA expression was normalized to small nucleolar RNA U6.

### Statistical analysis

Relative expression of miRNA was calculated using 2^-ΔΔct^ method in duplicate experiments (change fold = 2^−((Mean ΔCt Target)−(Mean ΔCt Calibrator))^) [24]. MicroRNA expression was normalized to endogenous control U6.

All values of miRNAs are expressed as mean ± SD. For categorical variables, the Chi-Square test was used. Independent samples t-test was used for 2-group comparisons. Differences were defined as statistically significant at a value of p < 0.05.

A composite score (denoted as miRNA-score) was defined to represent the cumulative levels of the miRNA (miR-LA, miR-SA, miR-CEmb and miR-UDN) in the ischemic stroke group compared with the control group as described previously [25]. The miRNA-score of each sample was calculated as the sum of the inverted-normalized signals of the miRNA and adjusted by subtracting a constant (the minimal score) so that the range of scores starts at 0 [25]. The ability to distinguish the ischemic stroke group and control group was characterized by the receiver operating characteristic (ROC) curve, and the area under the ROC curve (AUC) was calculated.

All statistical calculations were performed using SPSS 13.0 for Windows.

## Results

### Statistical analysis of patients’ characteristics

In our study, all patients suffered from ischemic stroke. Age, gender, smoking history, total triglyceride, total cholesterol, HDL, LDL, systolic blood pressure, diastolic blood pressure, and disease history were recorded. There were no significant differences among ischemic stroke groups and control group (p > 0.05). Details are shown in Table 1.

**Table 1:** Clinical characteristics of patients

***CHS***	***Age (YEARS)***	***M/F***	***CS (%)***	***TC***	***TG***	***HDL***	***LDL***	***SBP***	***DBP***	***DM***	***HT (%)***	***HL (%)***	***P***
**Stroke (24 h)**													
LA (n = 10)	62 ± 7	5/5	2 (20%)	4.48 ± 0.71	1.53 ± 0.43	1.13 ± 0.18	2.53 ± 0.58	132 ± 12	84 ± 13	1 (10%)	2 (20%)	2 (20%)	0.12
SA (n = 9)	63 ± 6	4/5	2 (22%)	4.32 ± 0.63	1.43 ± 0.23	1.24 ± 0.16	2.42 ± 0.61	125 ± 10	82 ± 14	2 (22%)	2 (22%)	1 (11%)	0.32
CEmb (n = 9)	64 ± 5	5/4	1 (11%)	4.18 ± 0.52	1.23 ± 0.18	1.02 ± 0.12	2.43 ± 0.47	130 ± 12	85 ± 11	1 (11%)	1 (11%)	1 (11%)	0.46
UDN (n = 10)	61 ± 6	5/5	2 (20%)	4.23 ± 0.34	1.36 ± 0.33	1.25 ± 0.27	2.45 ± 0.45	135 ± 13	80 ± 13	1 (10%)	2 (20%)	2 (20%)	0.78
**Stroke (1 W)**													
LA (n = 11)	65 ± 6	5/6	2 (18%)	4.12 ± 0.25	1.54 ± 0.46	1.15 ± 0.12	2.50 ± 0.35	128 ± 14	82 ± 10	1 (9%)	2 (18%)	2 (20%)	0.98
SA (n = 10)	66 ± 4	5/5	2 (20%)	4.16 ± 0.32	1.44 ± 0.35	1.10 ± 0.17	2.43 ± 0.42	138 ± 27	86 ± 14	1 (10%)	2 (20%)	1 (10%)	0.54
CEmb (n = 11)	62 ± 7	6/5	2 (18%)	4.31 ± 0.17	1.32 ± 0.38	1.15 ± 0.14	2.46 ± 0.38	137 ± 10	85 ± 13	1 (9%)	1 (9%)	1 (9%)	0.65
UDN (n = 10)	63 ± 7	4/6	2 (20%)	4.42 ± 0.54	1.42 ± 0.43	1.17 ± 0.12	2.35 ± 0.36	132 ± 15	87 ± 11	1 (10%)	1 (10%)	1 (10%)	0.98
**Stroke (4 W)**													
LA (n = 10)	65 ± 5	6/4	2 (20%)	4.39 ± 0.47	1.55 ± 0.39	1.18 ± 0.13	2.62 ± 0.56	130 ± 12	88 ± 10	1 (10%)	2 (20%)	2 (20%)	0.82
SA (n = 10)	64 ± 7	4/6	1 (10%)	4.36 ± 0.52	1.36 ± 0.36	1.09 ± 0.13	2.43 ± 0.34	129 ± 16	85 ± 11	2 (20%)	2 (20%)	1 (10%)	0.12
CEmb (n = 10)	63 ± 7	5/5	2 (20%)	4.26 ± 0.17	1.38 ± 0.29	1.12 ± 0.17	2.43 ± 0.33	130 ± 12	86 ± 10	1 (10%)	1 (10%)	1 (10%)	0.12
UDN (n = 10)	64 ± 6	6/4	2 (20%)	4.24 ± 0.43	1.42 ± 0.35	1.19 ± 0.16	2.44 ± 0.44	127 ± 13	82 ± 12	1 (10%)	2 (20%)	1 (10%)	0.34
**Stroke (24 W)**													
LA (n = 10)	65 ± 7	6/4	1 (10%)	4.36 ± 0.27	1.56 ± 0.42	1.14 ± 0.15	2.43 ± 0.56	126 ± 15	88 ± 12	1 (10%)	2 (20%)	2 (20%)	0.87
SA (n = 9)	66 ± 6	4/5	1 (11%)	4.39 ± 0.59	1.44 ± 0.32	1.17 ± 0.12	2.39 ± 0.45	124 ± 13	83 ± 11	2 (22%)	2 (22%)	1 (11%)	0.23
CEmb (n = 10)	64 ± 5	5/5	1 (10%)	4.35 ± 0.49	1.37 ± 0.41	1.15 ± 0.16	2.50 ± 0.43	126 ± 15	85 ± 12	1 (10%)	1 (10%)	1 (10%)	0.13
UDN (n = 9)	65 ± 6	5/4	1 (11%)	429 ± 0.39	1.49 ± 0.29	1.19 ± 0.18	2.45 ± 0.45	125 ± 13	86 ± 11	1 (11%)	1 (11%)	1 (11%)	0.56
**Stroke (48 W)**													
LA (n = 10)	64 ± 7	6/4	2 (20%)	4.46 ± 0.28	1.57 ± 0.42	1.15 ± 0.13	2.55 ± 0.43	127 ± 12	84 ± 12	1 (10%)	2 (20%)	2 (20%)	0.11
SA (n = 10)	66 ± 5	5/5	2 (20%)	4.35 ± 0.42	1.43 ± 0.32	1.17 ± 0.20	2.46 ± 0.53	123 ± 11	83 ± 10	2 (20%)	1 (10%)	1 (10%)	0.45
CEmb (n = 10)	65 ± 7	4/6	1 (10%)	4.37 ± 0.42	1.38 ± 0.41	1.23 ± 0.15	2.61 ± 0.53	125 ± 10	87 ± 14	1 (10%)	1 (10%)	1 (10%)	0.55
UDN (n = 9)	64 ± 8	5/4	1 (11%)	4.44 ± 0.32	1.42 ± 0.33	1.17 ± 0.18	2.65 ± 0.62	129 ± 13	87 ± 13	1 (11%)	1 (10%)	1 (10%)	0.87
**Healthy outpatient (n = 50)**	64 ± 6	24/26	10 (20%)	4.52 ± 0.48	1.39 ± 0.40	1.15 ± 0.14	2.50 ± 0.62	125 ± 11	82 ± 11	5 (10%)	5 (10%)	6 (12%)	

**CHS**, Characteristics; **M/F**, Male/female; **CS**, Current smoking; **TC**, total cholesterol (mmol/L); **TG**, total glyceride (mmol/L); **HDL**, high-density lipoprotein (mmol/L); **LDL**, low-densitlipoprotein (mmol/L); **SBP**, systolic blood pressure (mmHg); **DBP**, diastolic blood pressure (mmHg); **DM**, diabetes mellitus; **HT**, Hypertension; **HL**, Hyperlipidaemia; **LA**, Large artery stroke; **SA**, Small artery stroke; **CEmb**, Cardioembolic stroke; **UDN**, stroke due to undetermined cause; **P**, comparison between patients with healthy adult.

### MiRNAs plasma levels in ischemic stroke patients and healthy volunteers

**Figure 1:**
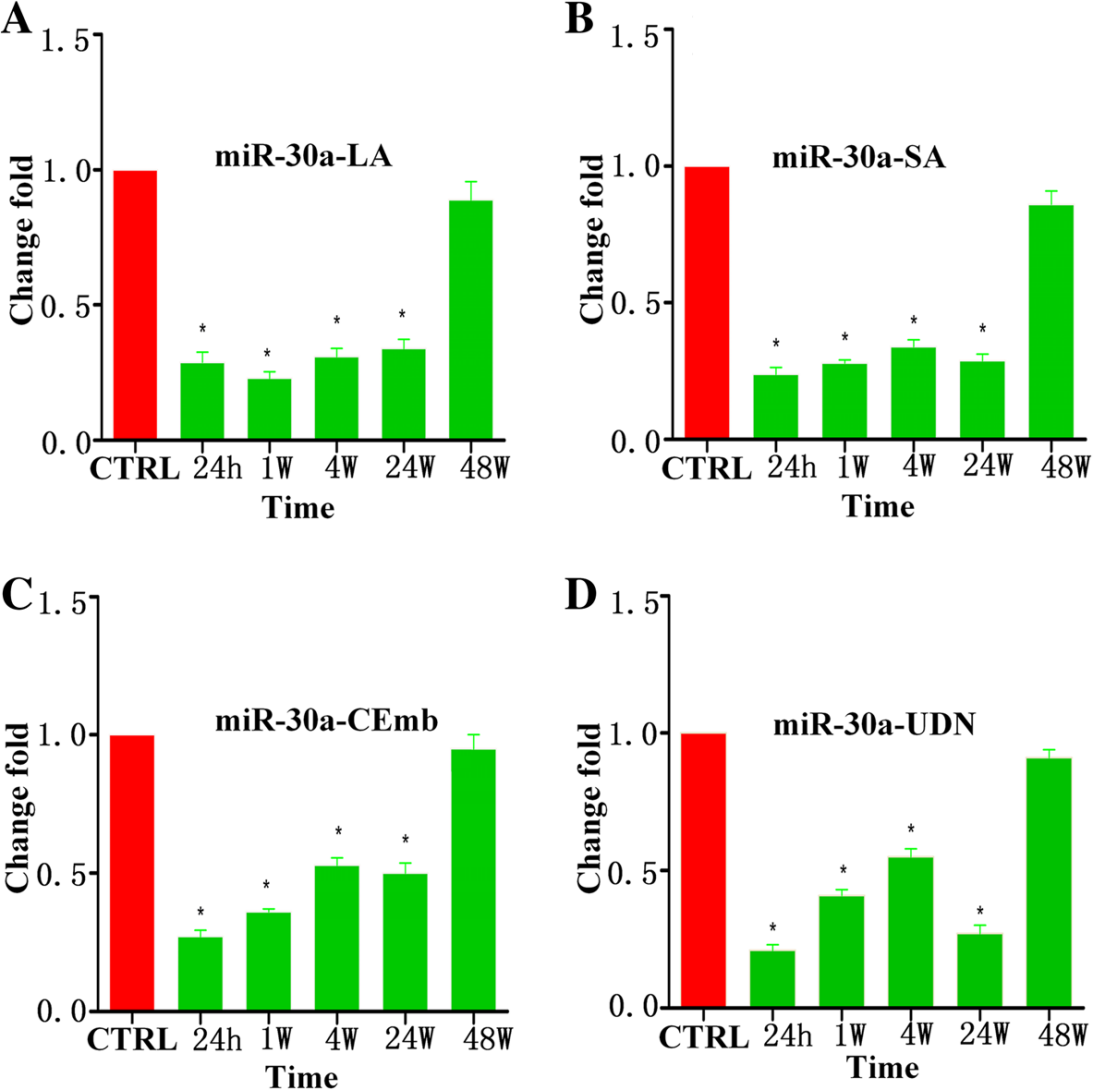
**Levels of miR-30a in plasma samples of patients with ischemic stroke at 24 h, 1 w, 4 w, 24 w and 48 w after the onset of symptoms. (A)** The levels of miR-30a-LA at different time points; **(B)** The levels of miR-30a-SA at different time points; **(C)** The levels of miR-30a-CEmb at different time points; **(D)** The levels of miR-30a-UDN at different time points (^*^ vs. control, p < 0.05).

**Figure 2:**
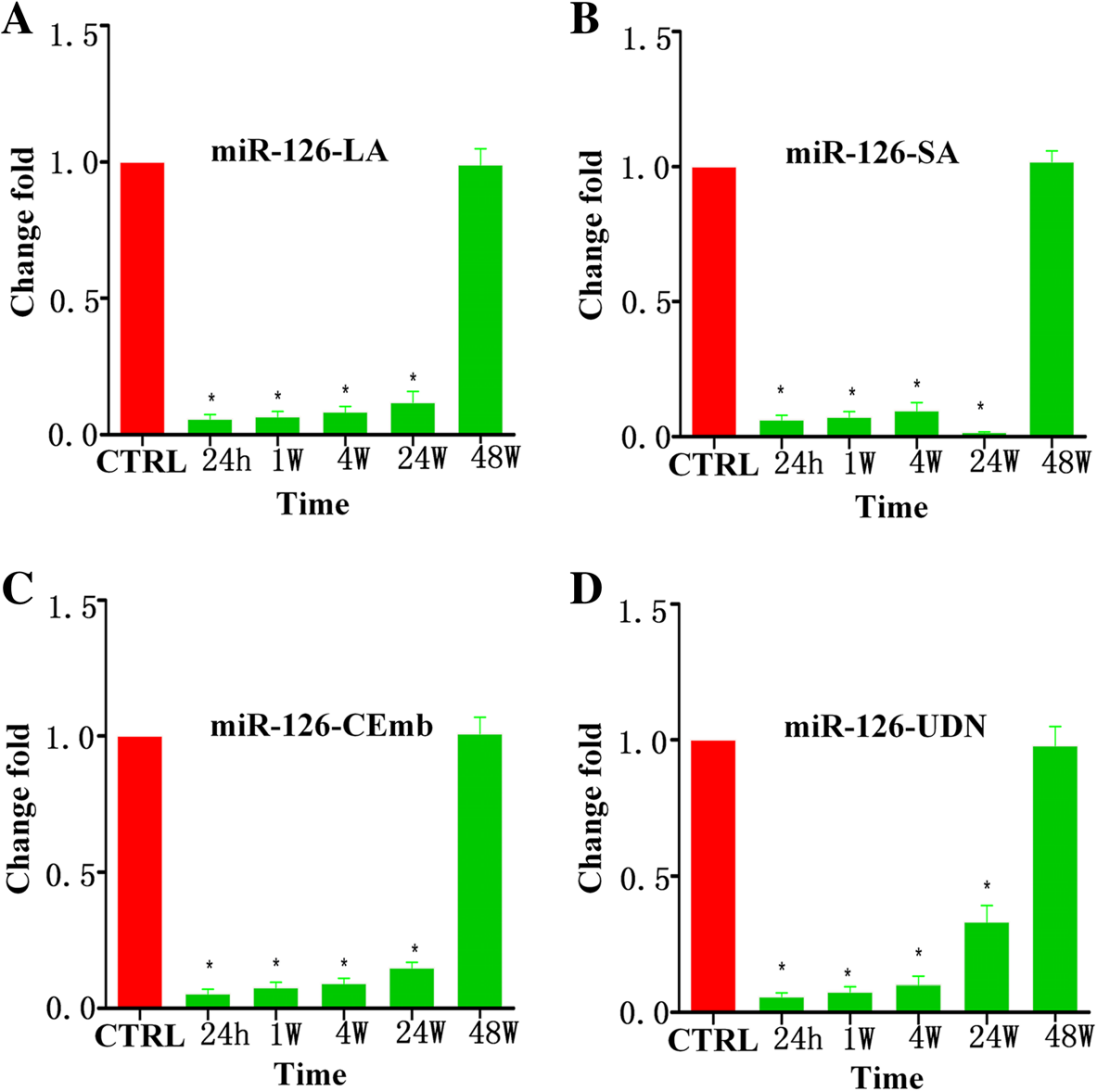
**Levels of miR-126 in plasma samples of patients with ischemic stroke at 24 h, 1 w, 4 w, 24 w and 48 w after the onset of symptoms. (A)** The levels of miR-126-LA at different time points; **(B)** The levels of miR-126-SA at different time points; **(C)** The levels of miR-126-CEmb at different time points; **(D)** The levels of miR-126-UDN at different time points (^*^ vs. control, p < 0.05).

**Figure 3:**
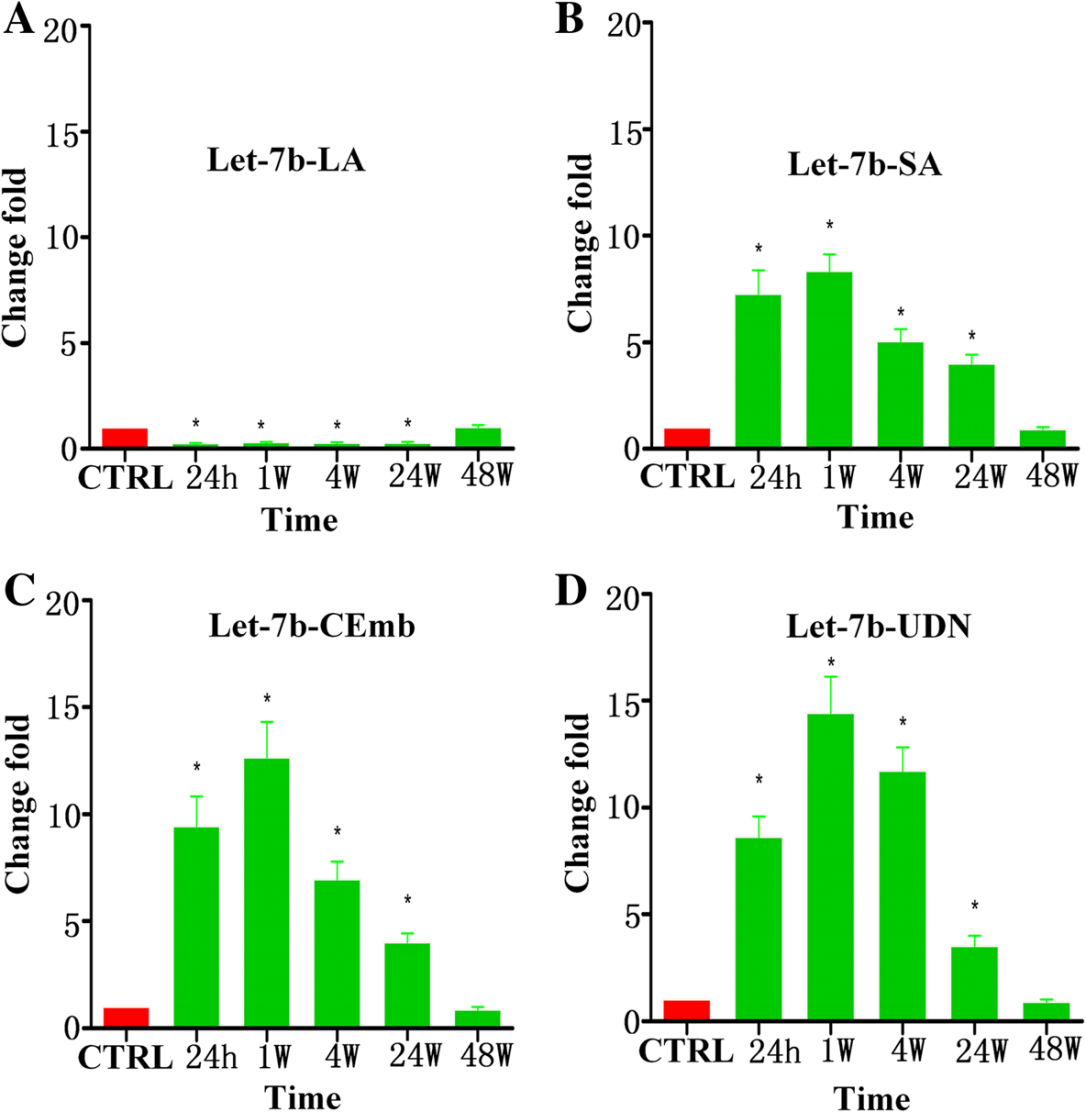
**Levels of let-7b in plasma samples of patients with ischemic stroke at 24 h, 1 w, 4 w, 24 w and 48 w after the onset of symptoms. (A)** The levels of let-7b-LA at different time points; **(B)** The levels of let-7b-SA at different time points; **(C)** The levels of let-7b-CEmb at different time points; **(D)** The levels of let-7b-UDN at different time points (^*^ vs. control, p < 0.05).

Using qRT-PCR assays, we measured the circulating levels of miR-30a, miR-126 and let-7b in ischemic stroke patients and healthy controls. Results are summarized in Table 2, Figures 1,2,3 and Additional file 1: Figure S1–S3. There were no significant differences among plasma miRNAs collected from patients and control at 48 w but it was found that circulating miR-30a and miR-126 were down-regulated in ischemic stroke patients at 24 h, 1 w, 4 w and 24 w. Plasma levels of miR-30a in all subtypes of ischemic stroke patients’ were 45%-79% lower than the controls at 24 h, 1 w, 4 w and 24 w (Figure 1). Plasma levels of miR-126 subtypes of ischemic stroke patients were 85%-98% lower than the healthy controls at 24 h, 1 w, 4 w and 24 w (Figure 2).

**Table 2:** Alterations in plasma miRNA levels in patients with ischemic stroke compared to healthy controls

	**STROKE**					**HA ΔCT ± SD**
	**24 h**	**1 w**	**4 w**	**24 w**	**48 w**	
**miR-30a**						−6.18 ± 1.27
**LA Δct ± SD**	−4.42 ± 0.57	−4.12 ± 0.43	−4.53 ± 0.46	−4.65 ± 0.47	−6.02 ± 0.45	
**SA Δct ± SD**	−4.15 ± 0.43	−4.36 ± 0.18	−4.63 ± 0.35	−4.38 ± 0.35	−5.97 ± 0.34	
**CEmb Δct ± SD**	−4.79 ± 0.40	−4.71 ± 0.15	−5.26 ± 0.26	−5.7 ± 0.42	−6.12 ± 0.32	
**UND Δct ± SD**	−3.94 ± 0.32	−4.91 ± 0.28	−5.33 ± 0.29	−4.29 ± 0.49	−6.05 ± 0.19	
**p value**	0.02	0.01	0.03	0.01	0.01	
**miR-126**						0.39 ± 0.012
**LA Δct ± SD**	4.51 ± 1.25	4.28 ± 1.13	3.95 ± 1.05	3.45 ± 1.03	0.41 ± 0.023	
**SA Δct ± SD**	4.38 ± 1.12	4.16 ± 1.15	3.75 ± 1.12	2.95 ± 0.23	0.36 ± 0.013	
**CEmb Δct ± SD**	4.57 ± 1.32	4.08 ± 0.98	3.86 ± 0.89	3.15 ± 0.43	0.37 ± 0.021	
**UND Δct ± SD**	4.49 ± 1.08	4.12 ± 1.02	3.69 ± 0.97	1.98 ± 0.38	0.42 ± 0.032	
**p value**	0.01	0.01	0.02	0.03	0.01	
**Let-7b**						−11.12 ± 2.16
**LA Δct ± SD**	−9.12 ± 1.25	−9.36 ± 1.06	−9.28 ± 1.34	−9.31 ± 1.13	−11.15 ± 1.22	
**SA Δct ± SD**	−13.98 ± 2.15	−14.18 ± 1.32	−13.46 ± 1.45	−13.12 ± 1.42	−11.01 ± 1.08	
**CEmb Δct ± SD**	−14.36 ± 2.18	−14.78 ± 1.95	−14.12 ± 1.53	−13.92 ± 1.67	−10.93 ± 1.52	
**UND Δct ± SD**	−14.23 ± 1.58	−14.97 ± 1.78	−14.67 ± 1.36	−12.93 ± 1.76	−10.96 ± 1.63	
**p value**	0.01	0.02	0.01	0.01	0.01	

Δct value of miR-126, miR-30a and let-7b in ischemic stroke groups and healthy adult. SD stands for the standard deviation of the average Δct of the group. Corresponding p values were calculated using the Independent-samples T test. HA Δct ± SD stands for Δct ± SD of healthy adult.

Interestingly, our data showed that the expression pattern of circulating let-7b in ischemic stroke patients with large-vessel atherosclerosis were different from patients with other subtypes. Specifically, plasma levels of let-7b from ischemic stroke patients with large-vessel atherosclerosis (let-7b-LA) were 70%-75% lower than in healthy controls at 24 h, 1 w, 4 w and 24 w (Figure 3A). However, it was highly expressed in other subtypes of ischemic stroke patients (let-7b-SA, let-7b-CEmb and let-7b-UDN) at 24 h, 1 w, 4 w and 24 w exhibiting 3.51-14.42 fold increase (Figure 3B-D).

Moreover, circulating levels of these miRNAs were also detected in blood samples from hemorrhagic stroke patients (Additional file 1: Figure S4). The results showed that the circulating levels of the three miRNAs did not change at the different time points examined.

### Specifity and sensitivity of miRNAs

Since the expressions of miRNAs may be affected by both technical and biological variation, we combined the levels of each miRNAs from different subtypes at the same time point into a single score to increase the signal to noise ratio. A miR-score represents the cumulative level of the miRNA (miR-LA, miR-SA, miR-CEmb and miR-UDN) for the comparison between ischemic stroke group and control group, which was described in the methods section. The miR-scores distinguished the ischemic stroke patients and the controls clearly (Table 3).

**Table 3:** Areas under the receiver operating characteristic curve and predictive value of three candidate miRNAs

	**95% CI**	**AUC**	**Cut-off point**	**Specificity (%)**	**Sensitivity (%)**
**miR-30a**					
**24 h**	0.869–0.979	0.91	1.675	94	80
**1 W**	0.848–0.971	0.91	1.75	93	84
**4 W**	0.856–0.976	0.92	1.67	90	84
**24 W**	0.875–0.984	0.93	1.665	92	84
**miR-126**					
**24 h**	0.871–0.978	0.92	1.75	92	84
**1 W**	0.895–0.985	0.94	1.875	90	86
**4 W**	0.878–0.982	0.93	1.845	92	84
**24 W**	0.864–0.977	0.92	1.77	92	82
**Let-7b**					
**24 h**	0.879–0.980	0.93	1.675	92	84
**1 W**	0.866–0.98	0.92	1.665	90	84
**4 W**	0.858–0.98	0.92	1.66	92	86
**24 W**	0.849–0.97	0.91	1.605	89	80

**Figure 4:**
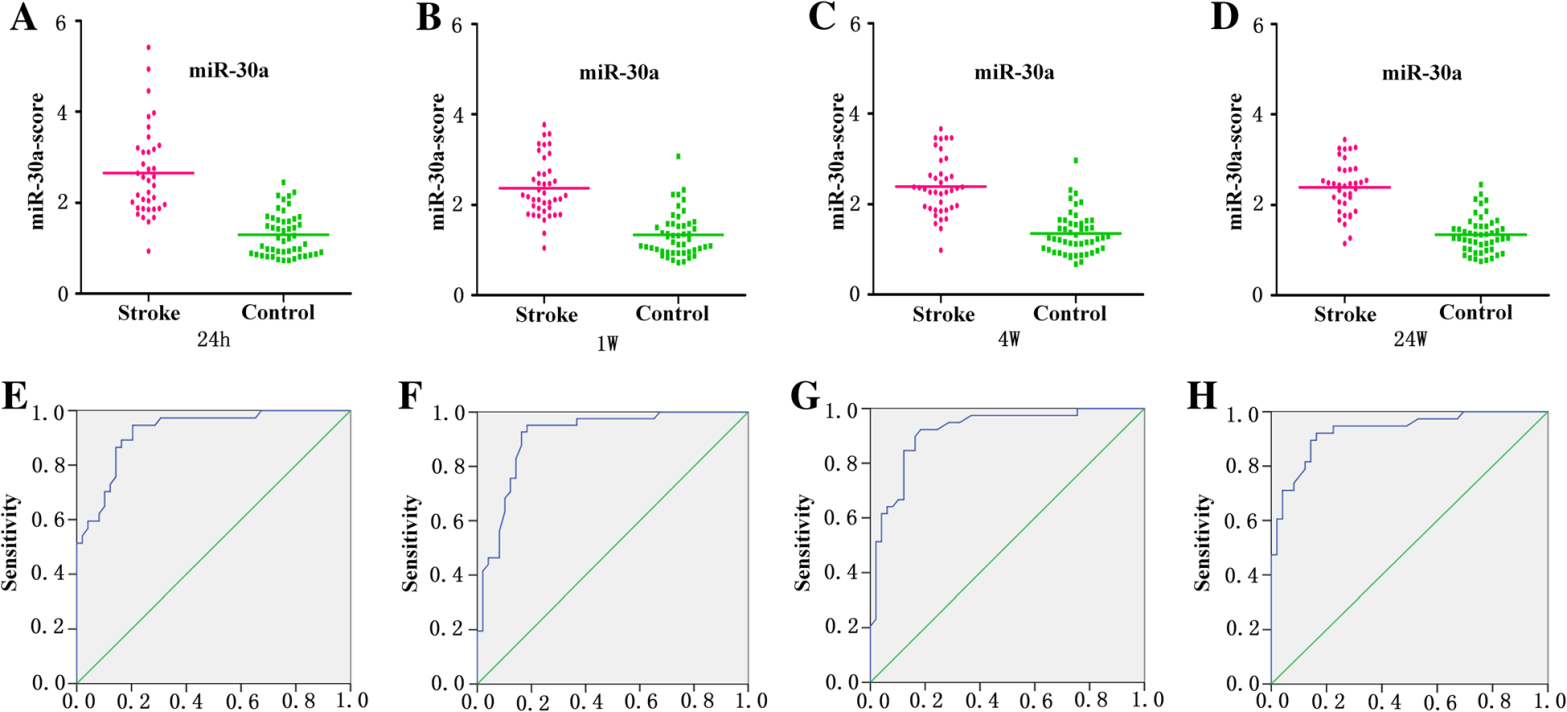
**Discrimination between ischemic stroke and control group at 24 h, 1 w, 4 w, 24 w using the composite miRNA score (miR-30a-score). (A-D)** The composite miR-30a-score was shown as median values in different groups. **(E-H)** ROC curve analyzed the diagnostic value of the composite miRNA-score.

The median score of miR-30a was 2.66, 2.37, 2.39 and 2.39 in stroke group and 1.30, 1.34, 1.35 and 1.34 in the control group at 24 h, 1 w, 4 w and 24 w, respectively (Figure 4A-D). The ability of the miR-30a-score to differentiate the stroke group from the control group was revealed further by the ROC curve with an AUC of 0.91 (95% confidence interval (CI) = 0.869-0.979), 0.91 (95% CI = 0.848-0.971), 0.92 (95% CI = 0.856-0.976) and 0.93 (95% CI = 0.875-0.984) at 24 h, 1 w, 4 w and 24 w, respectively. Using the optimal cutoff values of 1.675, 1.75, 1.67 and 1.665 for the diagnosis of stroke, we obtained a sensitivity of 94%, 93%, 90% and 92% and a specificity of 80%, 84%, 84% and 84% at 24 h, 1 w, 4 w and 24 w, respectively (Figure 4E-H).

**Figure 5:**
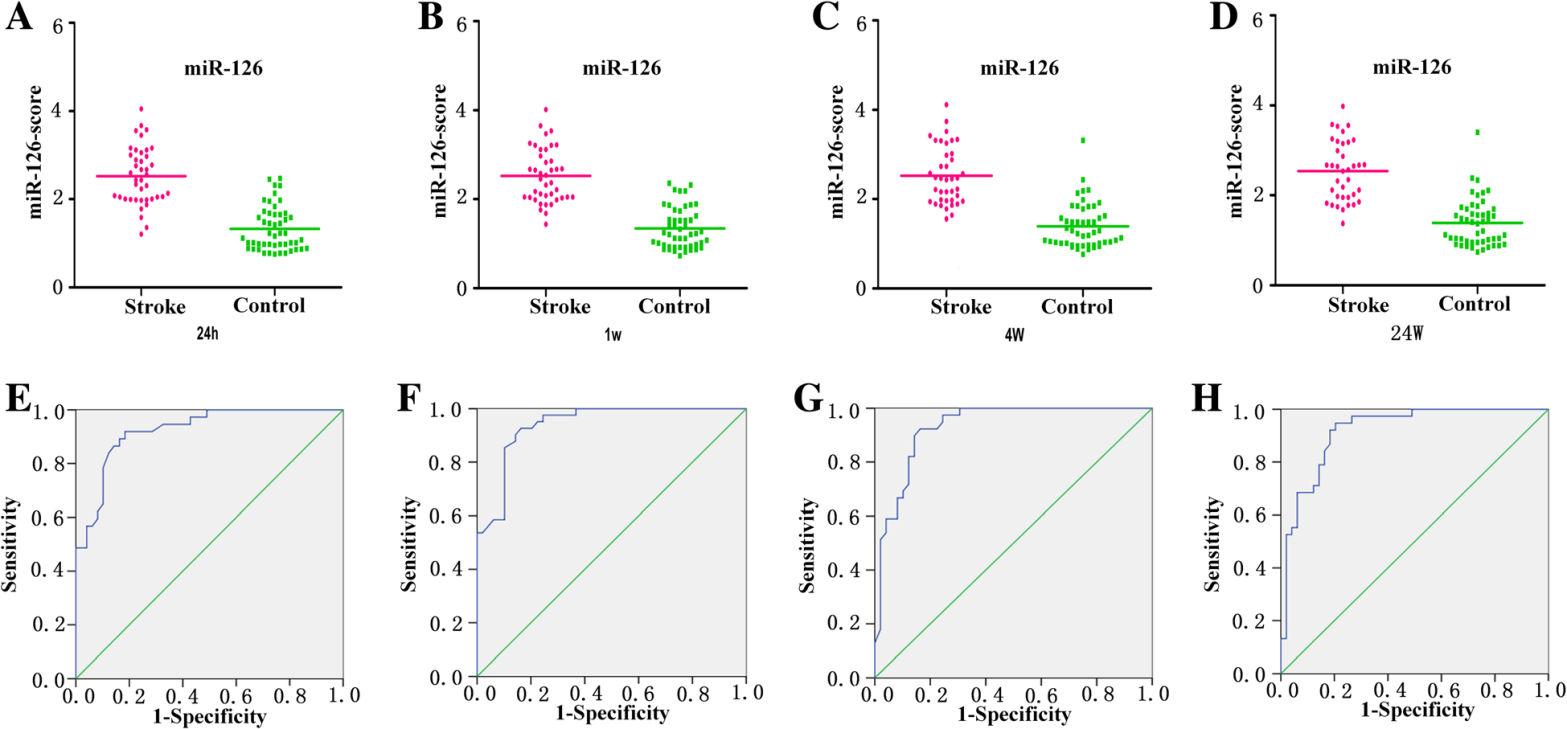
**Discrimination between ischemic stroke and control group at 24 h, 1 w, 4 w, 24 w using the composite miRNA score (miR-126-score). (A-D)** The composite miR-126-score was shown as median values in different groups. **(E-H)** ROC curve analyzed the diagnostic value of the composite miRNA-score.

When a comparison was made between the stroke patients and the healthy controls, the median score of miR-126 at 24 h, 1 w, 4 w and 24 w was 2.54, 2.52, 2.52 and 2.54, respectively in the stroke group, compared with 1.33, 1.35, 1.40 and 1.39 in the control group, respectively (Figure 5A-D). And the ROC curves with an AUC were 0.92 (95% CI = 0.871-0.978), 0.94 (95% CI = 0.895-0.985), 0.93 (95% CI = 0.878-0.982) and 0.92 (95% CI = 0.864-0.977). Using the threshold score of 1.75, 1.875, 1.845 and 1.77, the sensitivity of miR-126-score for the diagnosis of stroke was 92%, 90%, 92% and 92%, and the specificity was 84%, 86%, 84% and 82% at 24 h, 1 w, 4 w and 24 w, respectively. (Figure 5E-H).

**Figure 6:**
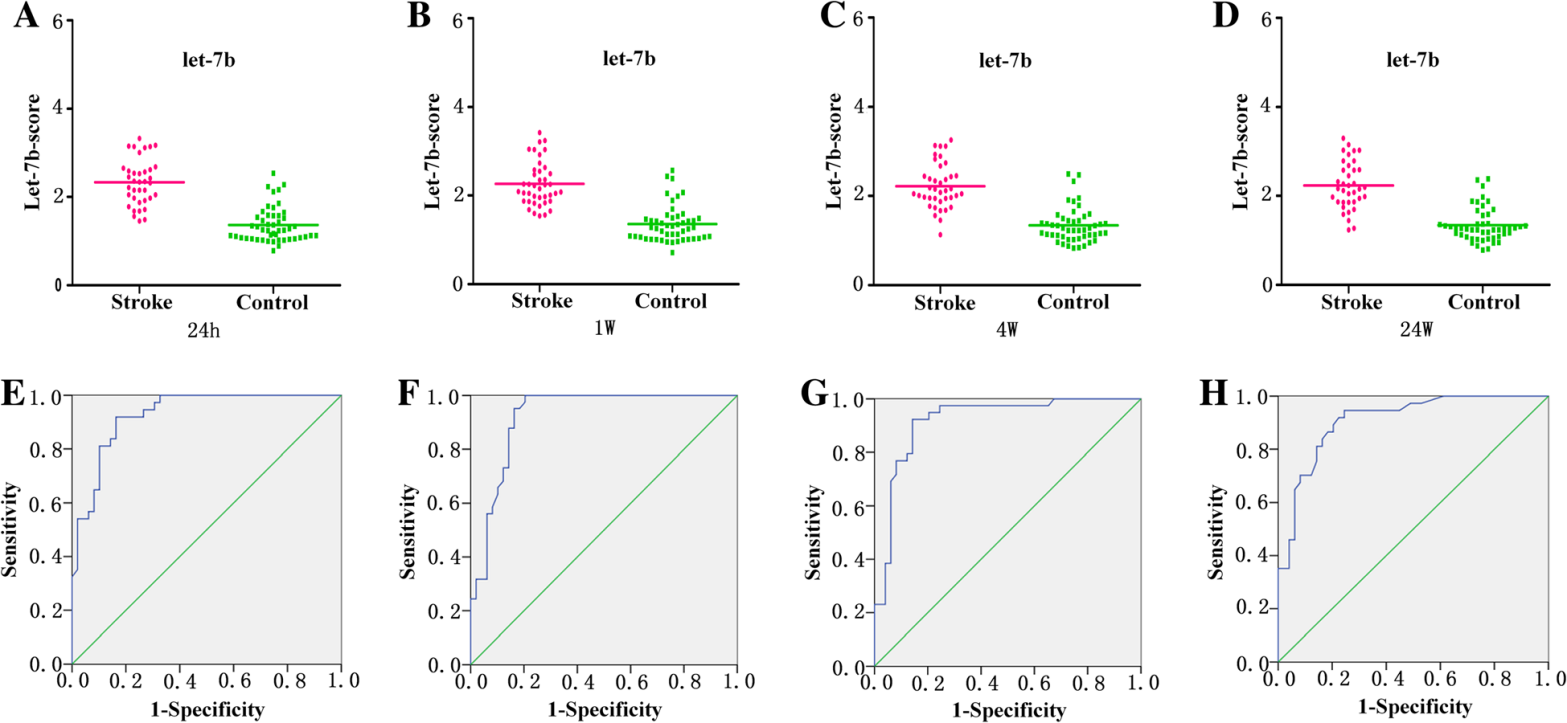
**Discrimination between ischemic stroke and control group at 24 h, 1 w, 4 w, 24 w using the composite miRNA score (let-7b-score). (A-D)** The composite let-7b-score was shown as median values in different groups. **(E-H)** ROC curve analyzed the diagnostic value of the composite miRNA-score.

Finally, the median score of let-7b was 2.33, 2.26, 2.22 and 2.23 in the stroke group, and 1.36, 1.35, 1.343 and 1.34 in the control group at 24 h, 1 w, 4 w and 24 w, respectively (Figure 6A-D). The ability of the let-7b-score to distinguish stroke group from control group was shown by the ROC curve with an AUC of 0.93 (95% CI = 0.879-0.980), 0.92 (95% CI = 0.866-0.98), 0.92 (95% CI = 0.858-0.98) and 0.91 (95% CI = 0.849-0.97). By using a threshold score of 1.675, 1.665, 1.66 and 1.605, above which patients were predicted to belong to the stroke group, we achieved a sensitivity of 92%, 90%, 92% and 89%, and a specificity of 84%, 84%, 86% and 80% for identification of ischemic stroke patients at the above mentioned time points. (Figure 6E-H).

## Discussion

At present, the diagnosis of stroke depends on clinical examination and various neuro-imaging techniques. However, there are no reliable circulating biomarkers for acute ischemic stroke risk prediction, diagnosis and outcome prediction [26,27]. It was reported that circulatory microRNA-145 expression is increased in cerebral ischemia, but the patient sample collection time was not mentioned [28]. In previous studies, we identified that circulating miRNAs were associated with human AMI [20-22]. In the present study, we found that the levels of circulating miR-30a, miR-126 and let-7b differed between the ischemic stroke and the control group. The most important findings are that the circulating levels of miR-30a, miR-126 and let-7b (let-7b-LA) were decreased and let-7b (let-7b-SA, let-7b-CEmb and let-7b-UDN) were increased in ischemic stroke patients than in the control group at 24 h, 1 w, 4 w, 24 w but not at 48 w.

It is widely believed that miRNAs released from damaged cells or circulating cells lead to increased plasma miRNA expressions [29]. However, the exact reason for miRNA levels decrease after ischemic stroke is not clear. We suspect that 1) circulating miRNAs are released by normal cells, and they are decreased when the cells are injured or cell density gets lowered; 2) circulating miRNAs are rapidly removed following their release into the circulation. However, further experiments are needed to explore these mechanisms.

To avoid possible bias from patients’ selection, individuals with similar age, gender, total cholesterol, HDL, LDL, triglyceride, systolic and diastolic blood pressures, diabetes and smoking status were recruited in the present study. And the statistical analysis implied that miR-30a, miR-126 and let-7b levels in plasma were not associated with those characteristics. These data further suggested that miR-30a, miR-126 and let-7b may be potential biomarkers for ischemic stroke.

The next questions are whether circulating levels of the plasma miR-30a, miR-126 and let-7b concentrations has any clinical significance and whether miRNAs might be a useful biomarker with diagnostic or prognostic roles in ischemic stroke patients. Using the levels of the three miRNA, we defined a score with a high specificity and sensitivity for the detection of ischemic stroke patients relative to control group. As a result, our data clearly verified the hypothesis that miR-30a, miR-126 and let-7b may be useful biomarkers for identifying the ischemic stroke.

Through bio-informatic analysis, we found that stroke related genes RhoB and beclin-1 might be the targets of miR-30a. Previously, miR-126 was reported to participate in atherosclerosis regulation by targeting 3′ UTR of VCAM-1 [30]. And let-7 family may be involved in stroke via inflammatory response [31]. It is important to consider the mechanisms by which these circulating miRNAs play a role in local pathophysiological processes. It has been shown that cell to cell communication can be mediated by exosomes containing miRNAs [32]. Recent data showed that miRNAs in extracellular vesicles facilitate communication between tumor cells and endothelial cells, and endothelial cells and smooth muscle cells, suggesting that stroke-related circulating miRNAs may be functional in the similar pattern [33,34].

It must be pointed out that this study has relatively small sample size and the results should be further validated in larger sample studies to confirm the role of miR-30a, miR-126 and let-7b levels as biomarkers for ischemic stroke in the future. Also, the sensitivity and specificity should be analyzed in larger, long term studies. And the reasons behind varying expressions of let-7b among various types of ischemic stroke deserves further investigation.

A cross-sectional study is an observational study in which exposure and outcomes are determined simultaneously for each subject. It is often described as taking a “snapshot” of a group of individuals. Cross-sectional studies are most appropriate for screening hypotheses because they require a relatively shorter time commitment and fewer resources to conduct [35]. However, there are some limitations of our study. Firstly, although we found that there is an association between circulating miRNAs expression and ischemic stroke, there is generally no direct evidence that the miRNAs caused the outcome. Secondly, ischemic stroke is not an inherent trait but one that developes over time, the causality of circulating miRNA is unclear. Finally, a follow-up study may be more helpful to better understand the association between circulating miRNAs expression and ischemic stroke.

## Conclusions

In summary, our data demonstrated a significant change in the circulating levels of miR-30a, miR-126 and let-7b in patients with ischemic stroke suggesting that miR-30a, miR-126 and let-7b might be the potential biomarkers for the diagnosis of ischemic stroke.

## Competing interests

The authors declare that they have no competing interests.

## Authors’ contributions

GL and FW carried out sample collection, RNA isolation, miRNA detection, performed the statistical analysis and drafted the manuscript. HL and ZY participated in sample collection, RNA isolation, miRNA detection. CS helped to revise the manuscript. YL participated in the data analysis. YW helped in collecting samples. CC conceived of the study, and participated in its design and drafted the manuscript. DWW participated in its design and coordination and helped to draft the manuscript. All authors read and approved the final manuscript.

## Pre-publication history

The pre-publication history for this paper can be accessed here:

http://www.biomedcentral.com/1471-2377/13/178/prepub

## Supplementary Material

Additional file 1: Figure S1 Levels of miR-30a in plasma samples of patients with ischemic stroke at 24 h, 1 w, 4 w, 24 w and 48 w after the onset of symptoms displayed by scatter. **(A)** The levels of miR-30a-LA at different time points; **(B)** The levels of miR-30a-SA at different time points; **(C)** The levels of miR-30a-CEmb at different time points; **(D)** The levels of miR-30a-UDN at different time points (* vs. control, p<0.05). **Figure S2.** Levels of miR-126 in plasma samples of patients with ischemic stroke at 24 h, 1 w, 4 w, 24 w and 48 w after the onset of symptoms displayed by scatter. **(A)** The levels of miR-126-LA at different time points; **(B)** The levels of miR-126-SA at different time points; **(C)** The levels of miR-126-CEmb at different time points; **(D)** The levels of miR-126-UDN at different time points (* vs. control, p<0.05). **Figure S3.** Levels of let-7b in plasma samples of patients with ischemic stroke at 24 h, 1 w, 4 w, 24 w and 48 w after the onset of symptoms displayed by scatter. **(A)** The levels of let-7b-LA at different time points; **(B)** The levels of let-7b-SA at different time points; **(C)** The levels of let-7b-CEmb at different time points; **(D)** The levels of let-7b-UDN at different time points (* vs. control, p<0.05). **Figure S4.** Levels of miRNAs in plasma samples of patients with ischemic stroke at 24 h, 1 w, 4 w, 24 w and 48 w after the onset of symptoms. **(A)** The levels of miR-30a at different time points; **(B)** The levels of miR-126 at different time points; **(C)** The levels of let-7b at different time points. **Table S1.** Patients’ functional status at the time of blood sampling. Click here for file
