# Supplementary material for: Circulating miR-30a, miR-126 and let-7b as biomarker for ischemic stroke in humans
Source: BMC Neurol. 2013 Nov 16;13:178. doi: 10.1186/1471-2377-13-178 (PMC3840584; doi:10.1186/1471-2377-13-178)
Supplement: Additional file 1: Figure S1 — Levels of miR-30a in plasma samples of patients with ischemic stroke at 24 h, 1 w, 4 w, 24 w and 48 w after the onset of symptoms displayed by scatter. (A) The levels of miR-30a-LA at different time points; (B) The levels of miR-30a-SA at different time points; (C) The levels of miR-30a-CEmb at different time points; (D) The levels of miR-30a-UDN at different time points (* vs. control, p<0.05). Figure S2. Levels of miR-126 in plasma samples of patients with ischemic stroke at 24 h, 1 w, 4 w, 24 w and 48 w after the onset of symptoms displayed by scatter. (A) The levels of miR-126-LA at different time points; (B) The levels of miR-126-SA at different time points; (C) The levels of miR-126-CEmb at different time points; (D) The levels of miR-126-UDN at different time points (* vs. control, p<0.05). Figure S3. Levels of let-7b in plasma samples of patients with ischemic stroke at 24 h, 1 w, 4 w, 24 w and 48 w after the onset of symptoms displayed by scatter. (A) The levels of let-7b-LA at different time points; (B) The levels of let-7b-SA at different time points; (C) The levels of let-7b-CEmb at different time points; (D) The levels of let-7b-UDN at different time points (* vs. control, p<0.05). Figure S4. Levels of miRNAs in plasma samples of patients with ischemic stroke at 24 h, 1 w, 4 w, 24 w and 48 w after the onset of symptoms. (A) The levels of miR-30a at different time points; (B) The levels of miR-126 at different time points; (C) The levels of let-7b at different time points. Table S1. Patients’ functional status at the time of blood sampling. [file 1471-2377-13-178-S1.doc]

**Figure S1**

**
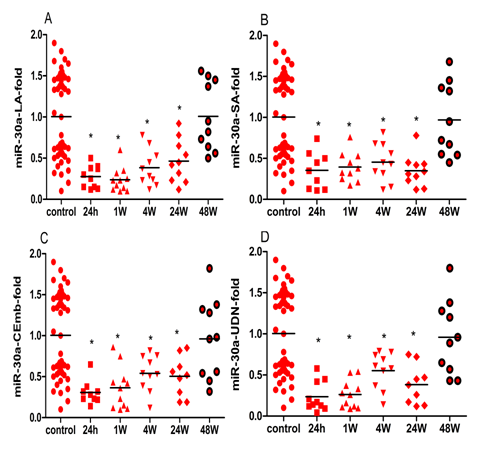
**

**Figure S1.** Levels of miR-30a in plasma samples of patients with ischemic stroke at 24h, 1w, 4w, 24w and 48w after the onset of symptoms displayed by scatter. (A) The levels of miR-30a-LA at different time points; (B) The levels of miR-30a-SA at different time points; (C) The levels of miR-30a-CEmb at different time points; (D) The levels of miR-30a-UDN at different time points (* vs. control, p<0.05).

**Figure S2**

**
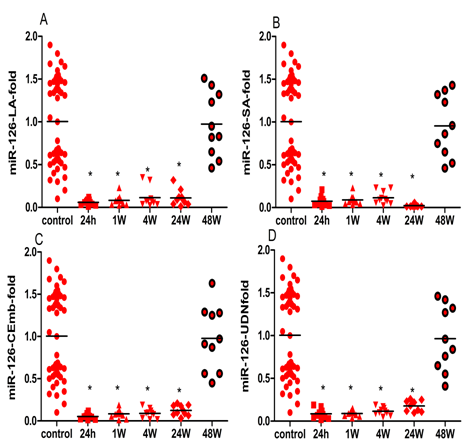
**

**Figure S2.** Levels of miR-126 in plasma samples of patients with ischemic stroke at 24h, 1w, 4w, 24w and 48w after the onset of symptoms displayed by scatter. (A) The levels of miR-126-LA at different time points; (B) The levels of miR-126-SA at different time points; (C) The levels of miR-126-CEmb at different time points; (D) The levels of miR-126-UDN at different time points (* vs. control, p<0.05).

**Figure S3**

**
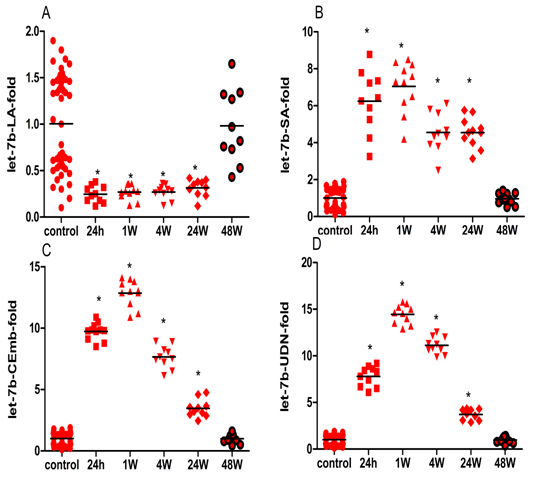
**

**Figure S3.** Levels of let-7b in plasma samples of patients with ischemic stroke at 24h, 1w, 4w, 24w and 48w after the onset of symptoms displayed by scatter. (A) The levels of let-7b-LA at different time points; (B) The levels of let-7b-SA at different time points; (C) The levels of let-7b-CEmb at different time points; (D) The levels of let-7b-UDN at different time points (* vs. control, p<0.05).

**Figure S4**


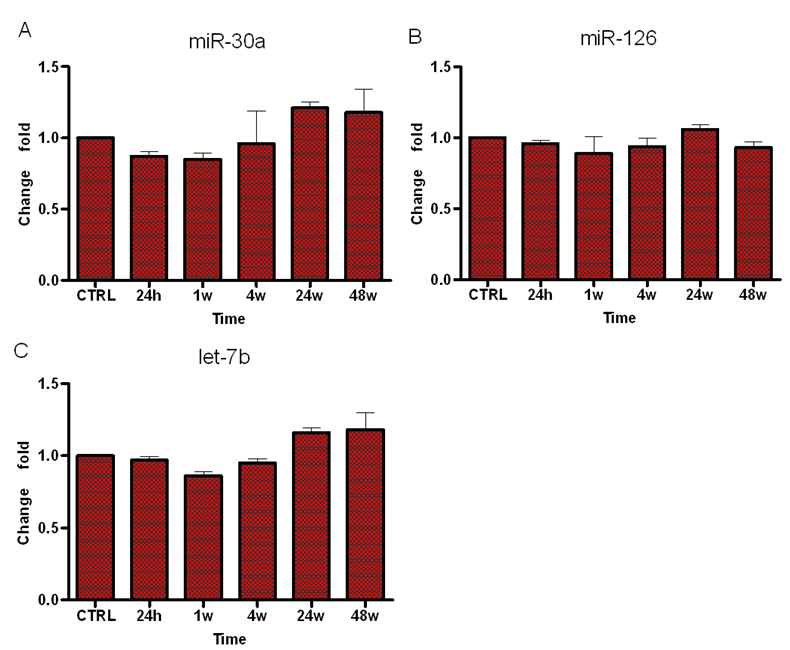


**Figure S4.** Levels of miRNAs in plasma samples of patients with ischemic stroke at 24h, 1w, 4w, 24w and 48w after the onset of symptoms. (A) The levels of miR-30a at different time points; (B) The levels of miR-126 at different time points; (C) The levels of let-7b at different time points.

**Table S1.** Patients’ functional status at the time of blood sampling.

| ***CHS*** | **MRS=2** | **MRS=3** | **MRS=4** | **MRS=5** |
| --- | --- | --- | --- | --- |
| **Stroke (24h)** |  |  |  |  |
| LA (n=10) | 3 | 1 | 3 | 3 |
| SA (n=9) | 1 | 3 | 2 | 3 |
| CEmb (n=9) | 1 | 2 | 3 | 3 |
| UDN (n=10) | 2 | 2 | 3 | 3 |
| **Stroke (1W)** |  |  |  |  |
| LA (n=11) | 2 | 3 | 3 | 3 |
| SA (n=10) | 1 | 3 | 3 | 3 |
| CEmb (n=11) | 2 | 3 | 3 | 3 |
| UDN (n=10) | 3 | 1 | 3 | 3 |
| **Stroke (4W)** |  |  |  |  |
| LA (n=10) | 2 | 3 | 3 | 2 |
| SA (n=10) | 2 | 3 | 3 | 2 |
| CEmb (n=10) | 2 | 2 | 3 | 3 |
| UDN (n=10) | 3 | 2 | 2 | 3 |
| **Stroke (24W)** |  |  |  |  |
| LA (n=10) | 2 | 3 | 3 | 2 |
| SA (n=9) | 3 | 1 | 3 | 2 |
| CEmb (n=10) | 3 | 2 | 2 | 3 |
| UDN (n=9) | 3 | 1 | 3 | 3 |
| **Stroke (48W)** |  |  |  |  |
| LA (n=10) | 2 | 2 | 4 | 2 |
| SA (n=10) | 2 | 3 | 2 | 3 |
| CEmb (n=10) | 3 | 2 | 2 | 3 |
| UDN (n=9) | 2 | 2 | 2 | 3 |

**mRS**, modified Rankin Scale; **LA**, Large artery stroke; **SA**, Small artery stroke; **CEmb**, Cardioembolic stroke; **UDN**, stroke due to undetermined cause.
